# Posttraumatic Soft Tissue Defects of the Heel: Comparison of Reconstructive Options and Results

**DOI:** 10.1055/s-0045-1812021

**Published:** 2025-10-10

**Authors:** Satyaki Roy, Harin Asokan, Jerry R. John, Ashu Rastogi, Satyaswarup Tripathy, Sunil Gaba, Ramesh K. Sharma

**Affiliations:** 1 Department of Plastic Surgery, Post Graduate Institute of Medical Education & Research, Chandigarh, India; 2 Department of Endocrinology, Post Graduate Institute of Medical Education & Research, Chandigarh, India

**Keywords:** heel pad avulsion, skin grafts, fasciocutaneous flaps, weight-bearing, reconstructive surgery, dynamic pedogram analysis, protective sensation, suprafascial versus subfascial, revision procedures, foot pressure distribution

## Abstract

**Introduction:**

The heel is difficult to reconstruct if it is avulsed off following accidents. Heelpad avulsion may be suprafascial or subfascial. Reconstructive options include skin grafts and flaps, depending on the involvement of weight-bearing area and exposed bone. We describe a series of patients who underwent heel pad reconstruction.

**Materials and Methods:**

Patients were grouped into two, depending on flap or graft reconstruction. The number of operations, time taken for recovery, complications, revision surgery, sensations, and footwear use were analyzed. Dynamic pedogram analysis was performed to assess heel pressures.

**Results:**

Twenty-one patients were assessed, out of which flap reconstruction was performed in 12. Nineteen patients achieved full weight bearing. Seventeen patients required revision procedures such as flap thinning. The mean time to walk, in the flap group was 14.3 weeks (range: 10–20 weeks) while that in the graft group was 11.5 weeks (range: 6–16 weeks). There was no difference in the reinnervation when comparing presence of light touch ( *p* = 0.49) and pain ( *p* = 0.37) between the two groups. On pedogram analysis, the mean peak pressures in the graft group were significantly less when compared with normal foot ( *p* = 0.017). The mean peak pressures were comparable in both involved and uninvolved feet, among patients who underwent flap reconstruction.

**Conclusion:**

Skin grafts demonstrated good stability even in the absence of customized footwear. A flap procedure can be avoided for suprafascial avulsions. The level of protective sensation achieved by spontaneous reinnervation seems to be sufficient for maintaining a functional well-healed foot.

## Introduction

The heel is a specialized anatomical area. Reconstructive techniques aim to replicate the thick, stable heel pad. Both the unique structure and the weight-bearing function have to be considered while reconstructing the heel. Apart from weight bearing, it also plays an important role in the walking cycle in the heel strike and heel rise phases. It helps maintain the balance of the body both during standing and walking.

Loss of the heel pad with exposure of tendon or bone presents problems because of a lack of adequate local tissue to provide cover. The relatively poor local vascularity and the weight-bearing needs compound this problem. 1 2 The results of suboptimal treatment can seriously impair the patient's quality of life. The ideal replacement for a heel defect should provide “anatomical contour, durable thick skin, protective sensibility, and stable soft tissue adherence to the underlying structures that can withstand the stress of ambulation.” 3 4 When subjected to an avulsing force, the skin and varying depths of the specialized subcutaneous tissue of the heel get detached from the underlying structures. Jeng et al. defined it a “suprafascial avulsion when the plane of cleavage is between the superficial and deeper subcutaneous layers.” It is called a “subfascial avulsion when the shearing plane is between the periosteum and plantar aponeurosis.” 4 The soft tissue flap of avulsed heel can be either proximal or distal based, complete or incomplete.

The present study aimed to compare the results of posttraumatic heel pad reconstruction with both skin grafts and flaps. The outcome was planned to be evaluated on the basis of the following parameters—time taken for return to daily activity; complications (early/late) at recipient site; dynamic foot pressure analysis and sensation (plantar surface, reconstructed area).

## Materials and Methods

This prospective observational study was conducted at a tertiary care center and included adult patients who presented with traumatic heel pad avulsion injuries requiring soft tissue reconstruction over a 2-year period. All patients were evaluated and treated based on injury characteristics and reconstructive needs.

Inclusion criteria were adult patients aged 18 years and above with isolated, traumatic heel pad avulsion injuries requiring surgical reconstruction. Patients who consented to participate and complied with postoperative follow-up were included. Exclusion criteria were bilateral heel pad injuries, associated tibial or fibular fractures, chronic wounds presenting more than 3 months' postinjury, and cases with bony malunion or nonunion in the affected limb.

Upon presentation, detailed clinical assessment was performed, classifying the avulsion injury based on the depth of tissue loss (suprafascial or subfascial), type of avulsion (proximal-based, distal-based, or complete), and whether tendon or bone was exposed. Reconstructive strategy was selected using a predefined algorithm ( Algorithm 1 ), guided by these classifications.

**Algorithm 1:**
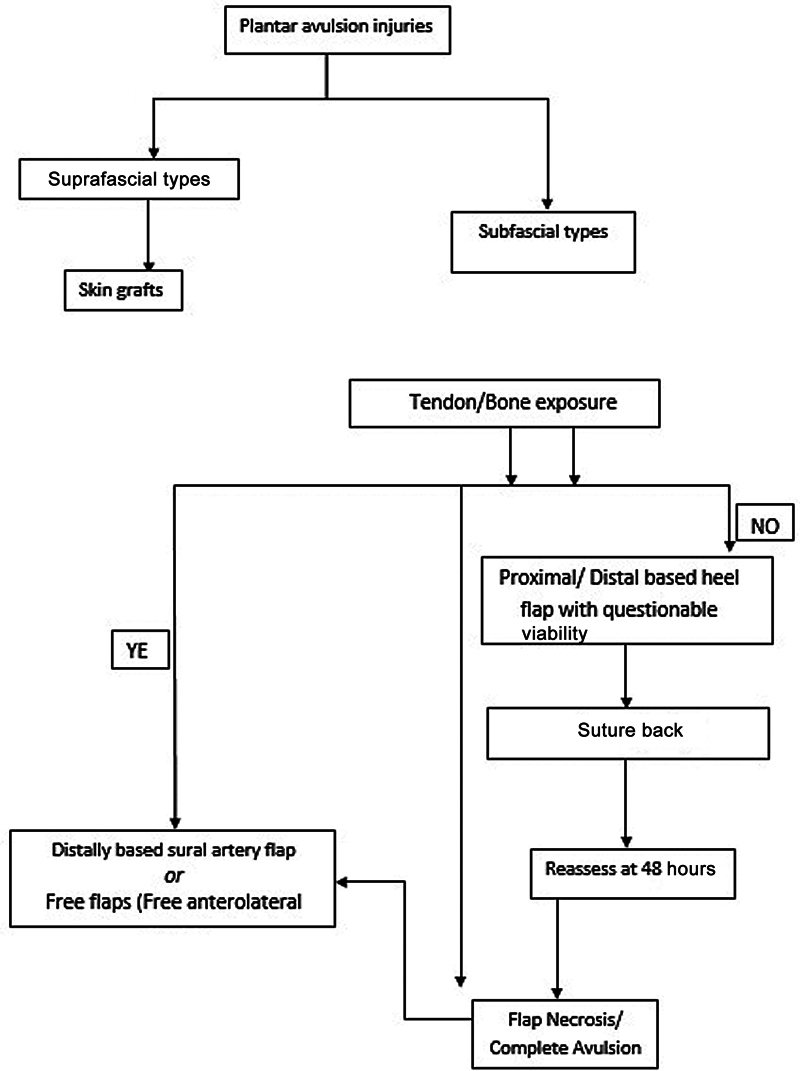
Reconstructive method was chosen based on the following algorithm.

When the avulsed tissue was viable, it was sutured back primarily. In cases where viability was questionable or there was significant tissue loss, the wound was reassessed after 48 hours. Suprafascial injuries without deep structure exposure were managed with split-thickness skin grafts. Subfascial avulsions or cases with exposed bone or tendon were reconstructed with flaps, including reverse sural artery (RSA) flaps, free anterolateral thigh (ALT) flaps, or other suitable options based on defect size and vascular status.

Postoperatively, skin grafts were inspected between the third and fifth day, whereas flap viability was monitored clinically by assessing color, capillary refill, turgor, and temperature. Patients were advised to avoid weight bearing on the reconstructed foot for a period of 3 to 6 weeks, until adequate healing occurred. Silicone heel pads were provided for all patients during the first 3 months to aid offloading. Customized footwear was recommended only when necessary, based on flap bulk, ulceration, or pressure mapping abnormalities. The patient was considered to be normally ambulant when full weight bearing was achieved for 5 hours on the reconstructed heel.

Patients were followed up for a minimum of 3 months. Outcome measures included time to full weight-bearing ambulation (defined as at least 5 hours per day), recovery of sensation (light touch, pain, and deep pressure), presence of early or late complications, need for revision procedures, and the type of footwear used. Revision procedures were offered to the patients depending on the presence of abnormalities in contour, ulceration, unstable skin, etc.

**Fig. 1:**
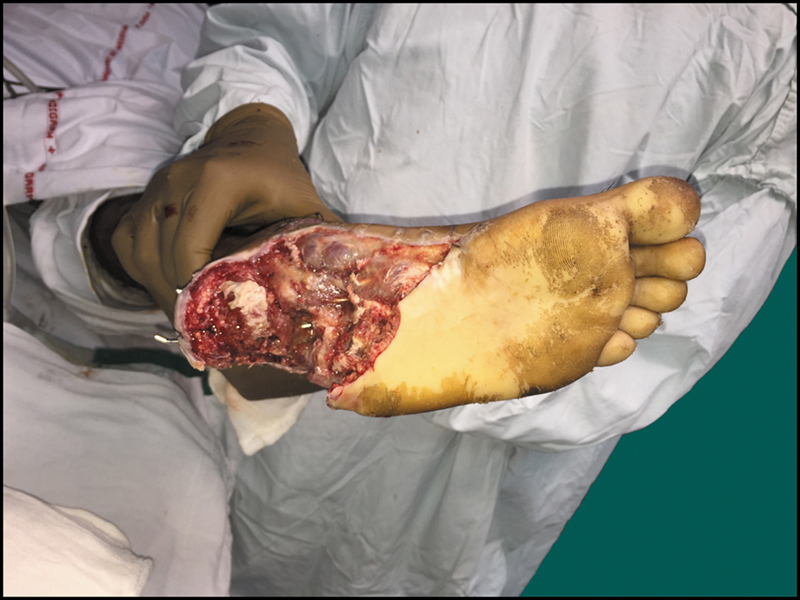
A 46-year man with complete left heel pad avulsion due to two-wheeler injury. The defect size was measured to be 9 × 11 cm.

Dynamic pedobarographic analysis was performed using dynamic foot pressure mapping to assess peak pressure and total contact area over both the reconstructed and contralateral normal foot. Foot pressure data (contact area and peak pressure) were collected using a platform sensor system through the “mid-gait” technique. The mid-gait technique requires that the patient walk across a walkway while pressure data are collected from a single foot contact over the sensor platform. The software divides the plantar surface of the foot into numerous regions to permit the analysis of data. The variables assessed are peak and average pressure, force, and area. Pressures were measured under the heel, midfoot, and forefoot. Peak pressures over corresponding areas of contralateral foot were also measured. Peak pressures and area of weight bearing were the variables assessed for comparison. Performance of the flaps and grafts was thus compared by a quantitative assessment of their weight-bearing characteristics during gait. Comparisons were made between patients' involved and uninvolved feet ( Figs. 1 2 3 4 5 ).

**Fig. 2:**
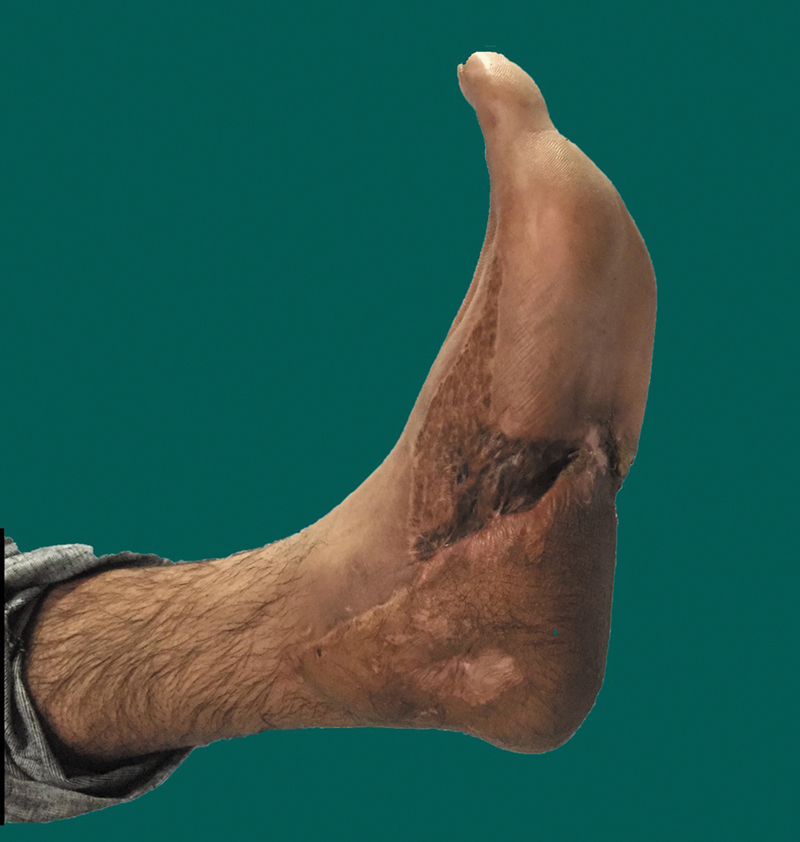
Clinical photo at 1-year follow-up of the same patient post ALT flap cover for the heel pad defect.

**Fig. 3:**
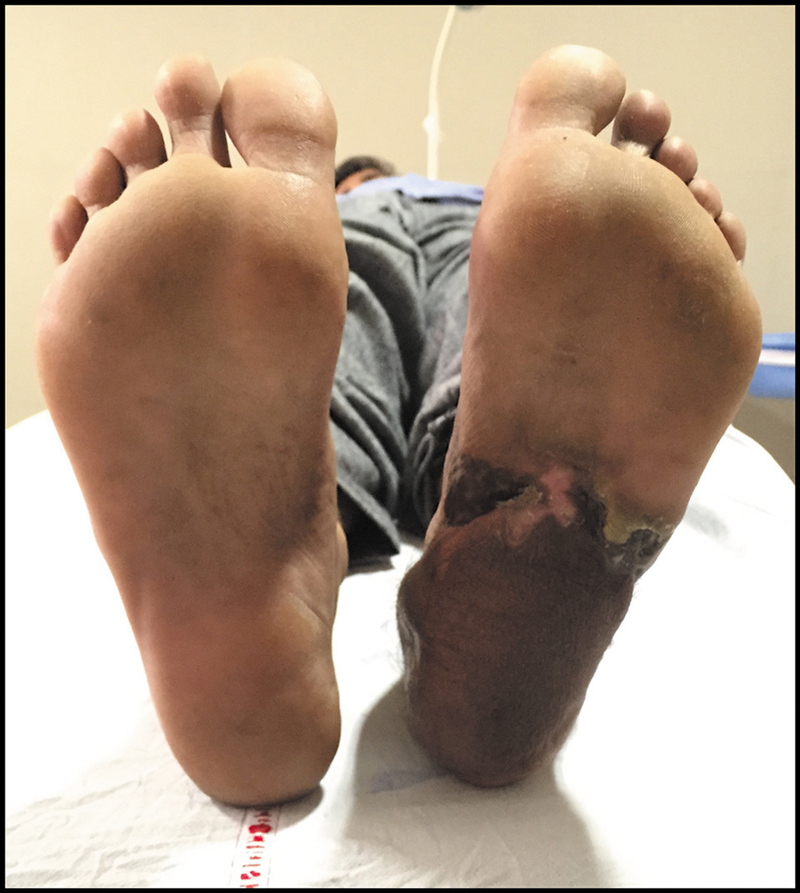
Comparison of reconstructed left heel pad with ALT defect and normal right heel pad.

**Fig. 4:**
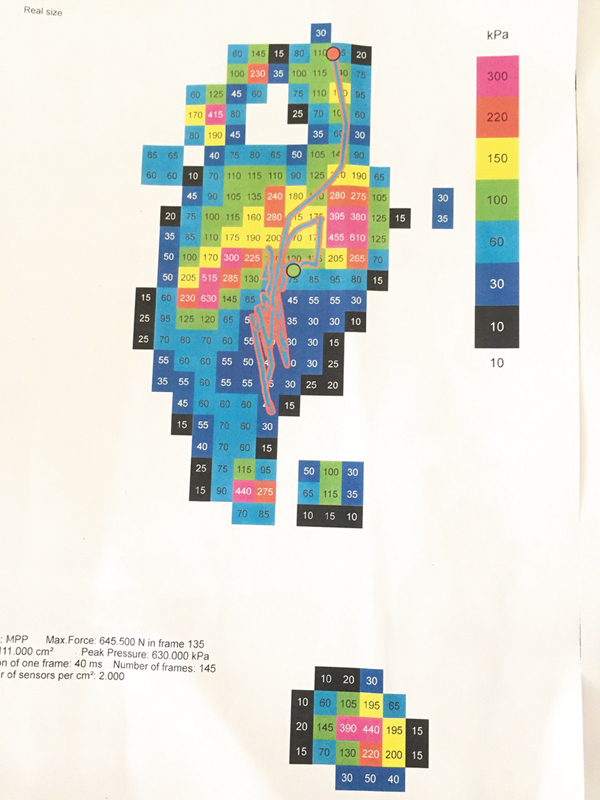
Podogram of left heel pad with ALT flap showing peak pressure of 630.00kPa.

**Fig. 5:**
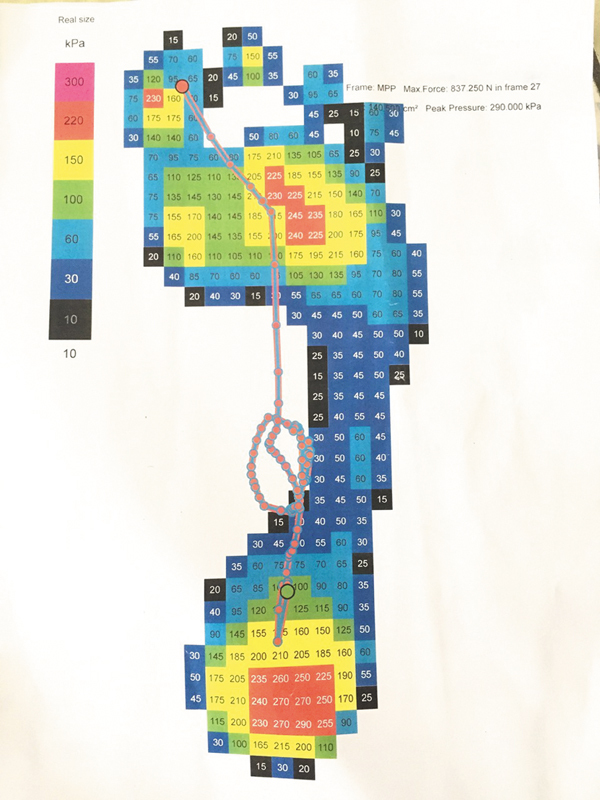
Podogram of right normal heel pad with peak pressure of 290.00 Kpa.

All data were analyzed using IBM Statistical Package for the Social Sciences (SPSS) Statistics version 22.0. Continuous variables were compared using Student's *t* -test or the Mann–Whitney U test, depending on normality of distribution. Categorical variables were analyzed using the chi-square test or Fisher's exact test where appropriate. A *p* -value of <0.05 was considered statistically significant Algorithm 1 .

## Statistical Analysis

The comparisons of domain scores and overall score in relation to type of surgery and time to walk were performed by using independent Student's *t* -test. The influence of type of surgery on revision procedures required was compared using Mann–Whitney *U* test. The association of type of surgery with sex of patient, the side and type of avulsion, use of customized footwear, ancillary support usage, early and late complications, and recovery of touch, pain, and pressure sensations were assessed by using χ ^2^ test or Fisher's exact test, whichever was applicable.

Peak pressure (skewed data) values over the reconstructed region obtained from pedogram analyses were compared by using Mann–Whitney *U* test, and Wilcoxon signed-ranks test was applied for time-related values. All statistical analyses were performed for two-tailed significance at 5% level of significance and *p* -value < 0.05 was considered as statistically significant. All analyses were done using IBM SPSS Statistics version 22.0.

## Results

Twenty-one patients were included in the study. Excluding a single patient who was lost to follow-up, 20 of them were subjected to statistical analysis ( Table 1 ). Thirteen were men and seven women. The mode of injury was traffic accident in all patients. Eighty percent of them involved two-wheelers. The male preponderance may reflect the comparatively commoner use of bikes among men.

**Table 1:** Patient background data

Case	Age (Yrs)	Sex	Side	Size (cm)	Depth	Surgery	Late complications	FU (Weeks)
1	23	M	Right	12 × 10	Subfascial	RSA flap	None	40
2	26	M	Right	13 × 10	Subfascial	ALT flap	None	54
3	50	M	Right	15 × 8	Suprafascial	SSG	None	39
4	11	F	Right	7 × 5	Suprafascial	SSG	Hypertrophic scar	58
5	25	F	Left	10 × 8	Subfascial	Cross leg flap	None	48
6	48	F	Left	6 × 5	Subfascial	SSG	Skin breakdown	35
7	36	M	Left	9 × 6	Subfascial	ALT flap	None	51
8	20	F	Right	4 × 4	Subfascial	LCA flap	None	50
9	40	M	Right	5 × 5	Suprafascial	SSG	None	36
10	50	M	Right	7 × 5	Subfascial	RSA flap	Trophic ulcer	53
11	55	F	Left	6 × 5	Suprafascial	SSG	None	65
12	34	M	Right	8 × 6	Subfascial	RSA flap	Skin breakdown	27
13	46	M	Left	11 × 9	Subfascial	ALT flap	None	52
14	42	M	Left	5 × 4	Suprafascial	SSG	Trophic ulcer	48
15	24	M	Right	7 × 6	Subfascial	RSA flap	None	28
16	26	F	Right	10 × 7	Subfascial	RSA flap	Hypertrophic scar	30
17	18	F	Left	12 × 6	Subfascial	SSG	None	22
18	23	M	Right	6 × 5	Suprafascial	SSG	Skin breakdown	32
19	30	M	Left	12 × 7	Subfascial	ALT flap	None	144
20	28	M	Right	10 × 8	Subfascial	ALT flap	Trophic ulcer	90

Twelve patients underwent a flap procedure while eight of them underwent split skin grafting. The two groups were comparable in terms of age, sex, side of reconstructed foot, depth, and type of avulsion. All patients of flap group had a subfascial avulsion as was decided in the initial treatment algorithm. However, two of the patients with subfascial avulsion were subjected to split skin grafting. For the first patient, this option was chosen because of paucity of local flap options and unavailability of a suitable donor vessel for free flap procedure. In the second, skin grafting was adopted as a temporary measure before definitive free flap cover. The patient, however, refused subsequent surgery in the follow-up visit.

Heel pad avulsions were assessed by reconstructive surgeon and classified as partial (less than 50% soft tissue loss) or complete (more than 50% heel pad soft tissue volume loss). Eleven patients had a complete avulsion of heel. Of the partial avulsions, six of them had a distally based flap and three of them, proximally based flaps. The lesser ratio of proximally based avulsions in our series reflects the increased survival of proximally based flaps, which were sutured back, as opposed to distally based ones, which had a greater likelihood of undergoing necrosis and subsequent reconstruction.

**Fig. 6:**
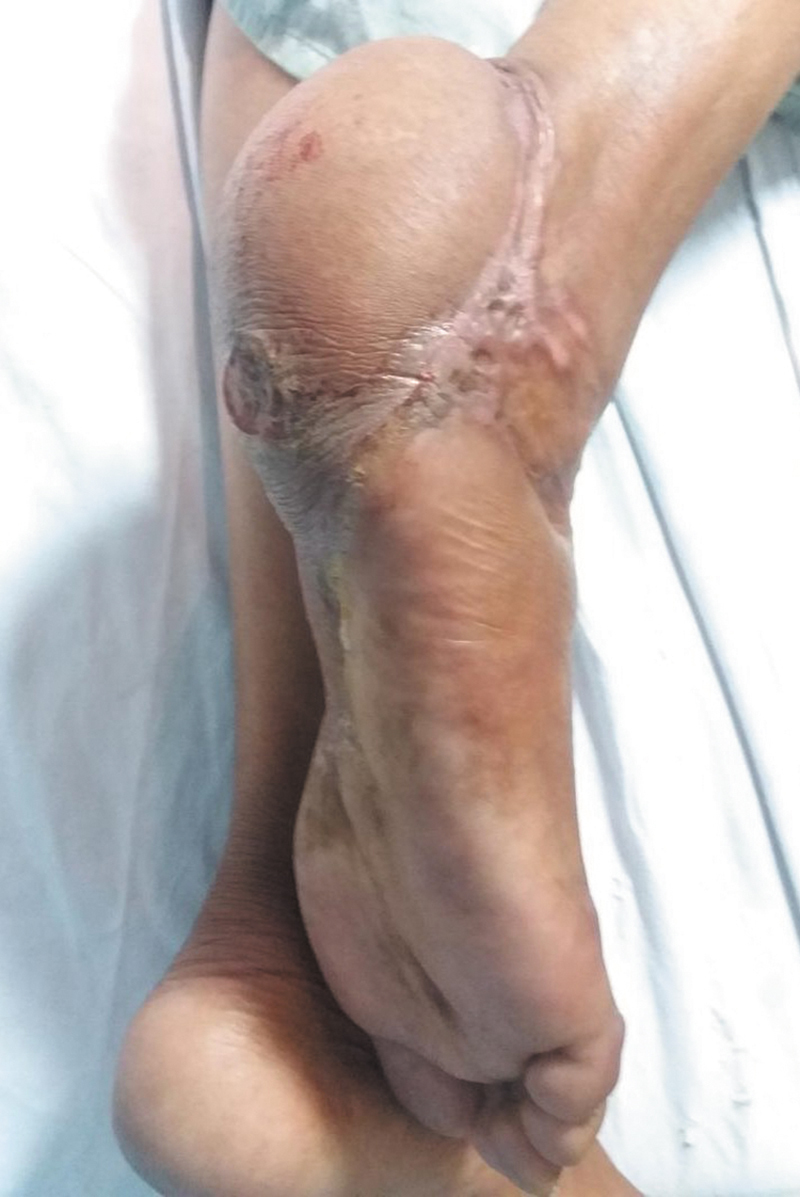
Trophic ulcer in a case with ALT flap cover over heel pad.

The 12 flap procedures performed included 5 RSA flaps, 5 free ALT flaps, 1 cross-leg flap, and 1 lateral calcaneal artery flap. The mean follow-up period was 42 weeks (range: 22–65 weeks) in the graft group and 56 weeks (range: 27–144 weeks) in the flap group. There was one instance of breakdown of local skin and three instances of trophic ulcers ( Fig. 6 ) among the patients who underwent flap coverage ( Table 2 ).

**Table 2:** Types of flaps with early and late complications

Flap group *N* = 12	Reverse sural artery flap (05)	Anterolateral thigh flap (05)	Cross Leg flap (01)	Lateral calcaneal artery flap (01)	Type of revision surgery
**Early Complications**					
Marginal flap necrosis	03	Nil	01	Nil	
Revision surgery	01	Nil	Nil	Nil	Debridement and SSG of necrosed margin
**Late complications**					
Trophic ulcer	02	Nil	01	Nil	Ulcer excision and closure
Skin breakdown	01	Nil	Nil	Nil	Nil

**Fig. 7:**
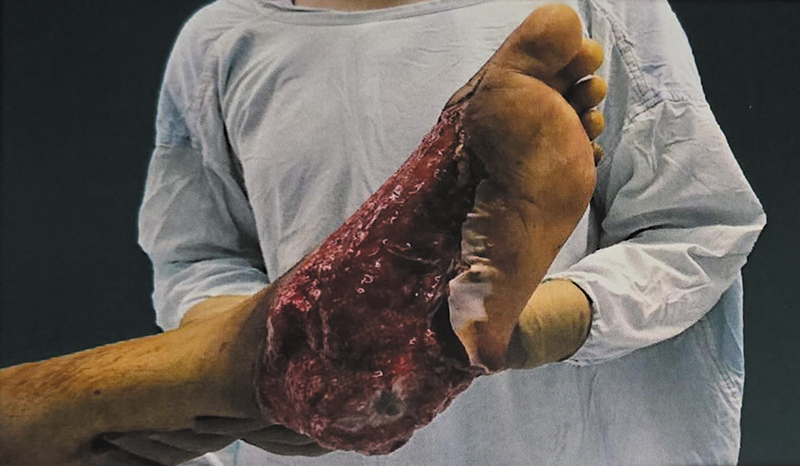
Heel pad avulsion injury over left foot.

**Fig. 8:**
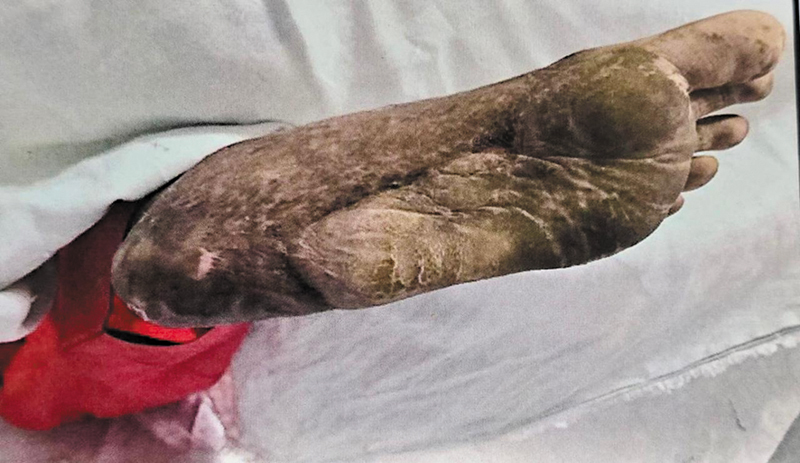
6 months follow-up for post grafting in heel pad.

Eight patients with heel pad injury underwent split skin grafting. ( Figs. 7 , 8 ). Two of the eight grafted patients suffered graft loss significant enough to warrant repeat grafting. Late complications in the skin grafted group included three trophic ulcers, three skin breakdowns, and two hypertrophic scars. All the trophic ulcers needed surgical intervention ( Table 3 ).

**Table 3:** Early and late complications in skin grafting group

Skin grafting group *N* = 08		
**Early complications**	No of cases	Revision surgery
Partial /significant graft loss	02	Repeat grafting
**Late complications**		
Trophic ulcer	03	Ulcer excision and closure
Skin break down	03	–
Hypertrophic scar	02	–

The incidence of complications was similar in both groups of patients. There was no difference among the two groups regarding the time taken to ambulation and the type of footwear (normal/custom) used and the number of revision surgeries performed.

The mean time to walk in the flap group was 14.3 weeks (range: 10–20), whereas that in the graft group was 11.5 weeks (range: 6–16). All patients used silicone heel padding for the initial 3 months. Only six patients were required to use customized footwear further. Three of them had trophic ulcers on their reconstructed region, two patients had recurrent skin breakdown, and one patient needed a larger size shoe to accommodate the bulk of the flap. Customized footwear was used to a similar extent by patients in both the groups ( *p* = 0.64).

There were 17 instances in which secondary surgery had to be performed. The procedures included, flap thinning in 12 patients, ulcer excision in 3 patients (one of them involved bony contouring in addition to ulcer excision), and repeat grafting in 2 cases ( Table 4 ). The difference in mean number of revision procedures between the flap and graft groups (1.17 vs. 0.38) was statistically significant ( *p* = 0.013).

**Table 4:** Revision surgeries performed

Case	Surgery	Number of revisions	Type of revision
1	RSA flap	2	Flap thinning
2	ALT flap	2	Flap thinning
5	Cross-leg flap	2	Flap thinning
7	ALT flap	2	Flap thinning
9	SSG	1	Repeat grafting
10	RSA flap	2	Flap thinning, ulcer excision
14	SSG	1	Ulcer excision
15	RSA flap	1	Flap thinning
16	RSA flap	2	Debridement and grafting of necrosed flap margin, flap thinning
17	SSG	1	Repeat grafting
20	ALT flap	1	Ulcer excision

Abbreviations: ALT, anterolateral thigh; RSA, reverse sural artery.

All patients used silicone heel pads for the first 3 months. This was found to reduce peak pressures over the sole on ambulation 2 5 and hence may prevent adverse pressure changes in the healing phase. Further, customized footwear was used in 25% of flap patients and 37.5% of grafted patients. No significant difference in use of such specialized footwear was noted between the two groups. Most of the modifications ( *n* = 5) were as flexible insoles which served to redistribute the pressure and offload the region of flap/graft. One of the flap patients required modifications to accommodate the increased bulk of the foot postreconstruction.

Two patients (one each in flap and graft group) reported use of a walking stick for support during ambulation. All the other patients in both groups were comfortable with full weight bearing in appropriate footwear. Patients with flaps on their heel also had hair growth on these flaps; this, however, was not found to hamper ambulation.

Sensations were found to be decreased in all reconstructed areas when compared with the normal foot. Although light touch was present only in two patients in the flap group ( *n* = 12), six of them recovered pain sensibility. All of them had deep pressure sensation. There was no difference in the reinnervation when comparing presence of light touch ( *p* = 0.49), pain ( *p* = 0.37) sensations between the two groups. Also, no significant difference was noted on comparing the recovery of light touch ( *p* = 0.35) and pain ( *p* = 0.37) sensations with occurrence of ulcer/skin breakdown in both groups.

On pedogram analysis, the mean peak pressure for all the patients who underwent a flap procedure was 301.25 kPa on the flapped heel. This was comparable to their normal heel mean peak pressure of 302.5 kPa. The mean peak pressure of all the patients who underwent split skin grafting was 241.25 kPa on the grafted heel. This was significantly less ( *p* = 0.017) than their normal heel mean peak pressure of 308.13 kPa. The peak pressures were comparatively lowest in the patients of subfascial avulsion who underwent split skin grafting.

The pressure analysis was also used to design off-loading insoles for patients who presented with ulcers and recurrent skin breakdown. We noted that such refinements led to early resolution of erosions, although ulcers required operative intervention.

## Discussion

The heel is a uniquely specialized structure, designed to endure high-impact forces. Its thick epidermis and dermis are tightly anchored to the underlying plantar aponeurosis by vertical fibrous septa, which not only resist shearing forces but also compartmentalize fat lobules that act as natural shock absorbers. This intricate architecture—comprising glabrous skin, fibrous septa, and interspersed fat—is unmatched by muscle, myocutaneous, or even fasciocutaneous flaps. Unfortunately, the availability of glabrous skin for reconstruction is minimal, limited primarily to the nonweight-bearing instep area supplied by the medial plantar artery. While this donor site is ideal for covering small defects like trophic ulcers, it falls short when addressing larger traumatic wounds. 6

The debate about the ideal tissue for heel pad replacement still remains unsettled. A skin graft may heal readily, but contraction may lead to restriction in joint motion. Fasciocutaneous flaps have dermis, subcutaneous tissue, and fascia in its entirety which protect against wound contracture as well as promote joint mobility due to the resultant elasticity. Muscle flaps undergo atrophy and fibrosis and may achieve a better contour when compared with fasciocutaneous flaps. The RSA flap is a reliable option for covering tendoachilles and heel defects. However, it lacks sensation and differs in texture from native plantar skin. Over time, although, the flap undergoes adaptive changes, and protective sensation tends to return gradually. 7 The present study was undertaken to assess outcomes in heel pad reconstruction, such as time taken to ambulate, early and late complications, return of protective sensations, and dynamic foot pressure analysis.

All patients included in the final analysis were ambulant at the time of the final assessment. The average time to full weight bearing in the flap group (14.3 weeks) was higher than that in the split skin graft (SSG) group (11.5 weeks). Few articles have reported time to walking with full weight bearing in their patient groups. 2 7 8 9 The time to ambulation in these studies varied from 7 weeks to 5 months. 8 10 The relatively longer time to normal ambulation noted in our study may stem from the more stringent definition (at least 5 hours of full weight bearing) for the outcome parameter as opposed to other studies, which report shorter durations. However, there is inadequate evidence in literature to compare this parameter between muscle and fasciocutaneous flaps.

Contrary to assumptions, skin grafts over subfascial avulsion defects ( *n* = 2) did not show any pressure changes. This may be because these patients are bearing less weight on the reconstructed foot, as objectively seen on pedogram analysis.

Long-term outcomes following heel reconstruction remain influenced more by functional integration than by the presence or absence of flap sensation. While sensate fasciocutaneous flaps may offer earlier recovery of light touch, studies have shown that nonsensate muscle flaps can perform equivalently in daily activities when protected appropriately. 11 Our findings reinforce this, demonstrating that spontaneous reinnervation—particularly deep pressure—was sufficient to restore protective sensation over time, regardless of the reconstructive method. Dynamic pedogram analysis further supports that pressure distribution normalizes in well-contoured flaps and even skin-grafted subfascial defects, challenging the necessity of sensate flaps in all cases. Instead, careful flap tailoring, offloading strategies, and patient adaptation appear to play a more decisive role in functional recovery than flap innervation alone.

The most common complication following heel pad reconstruction is ulceration. Although flap-related factors often play a role in its development, extrinsic factors like flap inset, recipient bed, and bony prominences may be contributory. 10 Potparic et al., while assessing durability of flaps over heel, noted that three of five recurrent ulcers developed in skin-grafted muscle flaps while two occurred in the fasciocutaneous flaps. 8 The anatomy of the underlying bone is a crucial factor leading to trophic ulcers. 5 10 Ozturk et al. showed that pressure spikes over palpable bone may be seen on podographic studies. 5 Ulcers occur because of bony injury, irregularity and ligamentous instability. Well-timed orthopaedic procedures may be adopted along with reconstructive surgery to minimize rates of ulceration. 9 12

There was a significant difference between rates of secondary interventions in skin graft and flap group. Almost all flaps underwent at least one sitting of flap thinning and contouring. It does appear that quality of flap tailoring and inset, and the nature of the recipient site determine the need for revision surgery, rather than factors pertinent to the flap or graft. This observation is in keeping with a previous study. 9 Adequate attention to removal of bony irregularity and proper tensioning of flap inset would reduce the need for revision surgery down the line.

Studies have reported that up to 60% of patients undergoing heel reconstruction with free flaps may require specialized footwear. 10 Surprisingly, majority of the patients with both flap and skin graft had pain-free ambulation with the use of normal footwear.

None of the flaps in our series underwent any type of reinnervation technique. Although deep pressure sensation was present in all patients, presence of pain and light touch sensations were variable. Many investigators have reported successful, durable coverage of the plantar surface after free muscle transfer and skin grafting, and have concluded that the presence of light touch sensation did not correlate with successful reconstruction. 13 Deep pressure sensation has been documented in both fasciocutaneous transfers without sensory nerve coaptation as well as in free muscle flaps covered with skin graft. 14 15 16

Durability of the reversed sural and free ALT flaps used in our series is apparent with regard to their stable wound cover. One of the patients who underwent reconstruction with an RSA flap and unwilling for further revision procedures required a larger size shoe. However, they were, in all cases except one, able to ambulate without inhibition. In contrast to our observations, some authors have reported that the high modulus of shear in the subcutaneous plane may cause instability while mobilizing. 17 18 19 20 21

On pedogram analysis, the peak pressures of flap patients in both feet were comparable. This is noted from the pedogram pictures, which showed comparable numbers in both the forefoot and hindfoot (over the flap) regions. This finding is in accordance with values published by Kuran et al. However, they also noted that patients with nonsensate flap reconstruction were reluctant to use their reconstructed foot and altered their gait in an attempt to reduce pressure over the loaded area of the reconstructed foot. 11 After heel reconstruction, patients transfer their weight to the forefoot. They tend not to press the reconstructed heel. In contrast, the peak pressures recorded by Karakostas et al. were two to three times more over the reconstructed area than normal foot. 22 They have postulated that this finding may be a reflection of decreased area of reconstructed part in contact with the ground. While we have assessed the outcomes in our study using various clinical parameters and pedogram analysis, outcomes can also be assessed using the subjective component of the American Orthopaedic Foot and Ankle Society hindfoot clinical rating scale, which has been shown to be reliable and valid. 23

The skin-grafted patients' group showed lesser peak pressures in the affected foot. The significantly lesser pressures in the graft group as compared with flap group may be a result of increased effective area of weight bearing leading to a more even pressure distribution. The difference was more glaring in the patients of subfascial avulsion who were subject to skin grafting. These patients may be loading the reconstructed region less by modification of their gait.

Skin substitutes have emerged as promising adjuncts in the management of chronic and complex wounds, offering scaffolding, growth factors, and cellular support to aid healing. 24 25 26 While several commercially available products have shown efficacy in ulcers and partial-thickness injuries, their role in weight-bearing areas such as the heel remains limited due to insufficient durability and poor mechanical resistance. Moreover, most skin substitutes lack the adherence and structural integration required to replicate the shock-absorbing function of the native heel pad. We did not consider skin substitutes in our treatment algorithm for the sake of uniformity of the study and to enable a focused comparison between flap and graft-based reconstructions in traumatic avulsion injuries.

We admit that technique-wise, the two groups (graft vs. flap) are heterogenous, and comparison is limited in scope. Still, optimal functional outcomes across surgical techniques need to be sought for, when a problem may be solved with more than one solution. We also recognize that an increased sample size would have helped to validate our findings.

## Conclusion

Both skin grafts and fasciocutaneous flaps can provide durable replacement of the weight-bearing heel. Skin grafts demonstrated good stability even in patients using normal footwear, and hence, a flap procedure may be avoided in suprafascial heel avulsions. Flap tailoring and bony contouring at the time of initial surgery go a long way in reducing the number of revision procedures required. The level of protective sensation achieved by spontaneous reinnervation seems sufficient for maintaining a functional well-healed foot.
